# Risk Factors of Anastomotic Leakage After Esophagectomy With Intrathoracic Anastomosis

**DOI:** 10.3389/fsurg.2021.743266

**Published:** 2021-09-21

**Authors:** Huan Li, Shimin Zhuang, Honghong Yan, Wenxiao Wei, Quanguan Su

**Affiliations:** ^1^State Key Laboratory of Oncology in South China, Department of ICU, Collaborative Innovation Center for Cancer Medicine, Sun Yat-sen University Cancer Center, Guangzhou, China; ^2^Department of Otolaryngology-Head & Neck Surgery, The Sixth Affiliated Hospital of Sun Yat-sen University, Guangzhou, China

**Keywords:** esophageal cancer, esophagectomy, intrathoracic anastomotic leakage, postoperative hospital stay, risk factor

## Abstract

**Purpose:** Anastomotic leakage is one of the most common complications of esophagectomy, it serves as one of the main causes of postoperative death of esophageal cancer. It is of clinical significance to try to discover the risk factors that cause anastomotic leakage.

**Methods:** This retrospective study was conducted on 1,257 consecutive esophageal cancer patients who underwent esophagectomy with intrathoracic anastomosis from January 2010 to December 2015 at a high volume cancer center. Multivariate Logistic Regression analysis, Spearman rank correlation analysis, Mann-Whitney U test and Kruskal-Wallis test were performed to identify the risk factors to the occurrence of anastomotic leakage and the length of hospital stay.

**Results:** Intrathoracic anastomotic leakage occurred in 98 patients (7.8%). Older patients were more likely to develop anastomotic leakage. Patients with diabetes had a higher leakage rate. Intrathoracic anastomotic leakage, old age as well as comorbidities were associated with longer hospital stay.

**Conclusion:** Our study suggested that old age and diabetes were risk factors to intrathoracic anastomotic leakage. In-hospital stay would be lengthened by intrathoracic anastomotic leakage, old age and comorbidities.

## Introduction

Esophageal cancer is a common malignant tumor of the alimentary tract (1). Surgical resection is still the main method for the treatment of early and middle stage of esophageal cancer. Although surgical techniques have made great progress, (2) radical resection of esophageal cancer remains very traumatic and complicated, and there are many complications in the perioperative period.

Anastomotic leakage is one of the most common and serious complications of esophagectomy, it serves as one of the main causes of postoperative death after esophageal cancer operation (3–5). Therefore, early diagnosis and early treatment of anastomotic leakage is of much importance to reduce the mortality after esophageal cancer operation.

Some studies have found that the occurrence of anastomotic leakage is related to a variety of factors, such as age, histological cell type of tumor, the site of anastomosis, tumor staging, etc (3, 6–8). But no clear conclusions could be drawn up to now. There are significant differences in the incidence, severity, clinical manifestation, and prognosis of intrathoracic anastomotic leakage and cervical anastomotic leakage (9, 10). But so far, few articles focused on the influential factors of intrathoracic anastomotic leakage in esophageal cancer patients have been reported.

Esophageal cancer is one of the most common malignant tumors in China, and squamous cell carcinoma is the dominant histologic type. This is obviously different from western countries, where adenocarcinoma is most commonly seen (11). Therefore, the risk factors affecting anastomotic leakage may be different between China and western countries. But as far as we know, few reports of risk factors for intrathoracic anastomotic leakage in Chinese esophageal cancer patients have been published.

Moreover, even less articles concerning the associated factors to the length of postoperative hospital stay in esophagectomy patients have been reported. Since esophageal cancer resection is a high-risk surgical procedure, searching for the influential factors with the length of hospital stay may be helpful to improve the clinical outcomes.

The objective of this study is to investigate the associated factors with intrathoracic anastomotic leakage, as well as the risk factors to the length of hospital stay after esophagectomy in Chinese esophageal cancer patients at a high volume cancer center.

## Materials and Methods

Totally 1,284 patients had undergone esophagectomy with intrathoracic anastomosis from January 2010 to December 2015, in the thoracic surgery department of Sun Yat-sen University Cancer Center. The vast majority of them underwent Sweet procedure, and only 27 cases (2.1%) underwent Ivor Lewis procedure. In order to have a more homogeneous population, only those 1,257 cases with Sweet procedure were enrolled in this study. Sweet procedure is a surgical operation in which esophageal cancer is removed through a left posterolateral thoracic incision and the stomach replaces the esophagus. It is a standard operation for esophageal cancer, especially for lower esophageal cancer, which has the advantages of simplicity, convenience and high success rate. All patients were sent to the intensive care unit (ICU) after operation. Anastomotic leakage was observed closely during the follow-up period. If any signs of leakage (e.g., fever or chest pain, palpitation, shortness of breath, dyspnea, a significant increase in blood leucocyte, pleural effusion or pneumothorax, gastrointestinal contents found in thoracic drainage tube) appeared, further examination such as water-soluble contrast swallow study, endoscopy or CT scan could be performed to confirm the presence of leakage.

Relevant clinical and pathological data including age, gender, location of tumor, histological subtype, history of comorbidities such as hypertension and diabetes, pathological TNM stage were obtained retrospectively from medical records. The comorbidities in this study referred only to hypertension or/and diabetes. All the cases included in this study had normal liver and kidney function before surgery, and no case was accompanied with cirrhosis. The tumors were staged according to the seventh edition of the Union for International Cancer Control (UICC) TNM staging system. Postoperative information such as the leakage of anastomosis, length of hospital stay was also collected. “Total hospital stay” was defined as “the total number of days from admission to discharge;” and “Postoperative hospital stay” was defined as “the number of days from surgery to discharge.”

Continuous variables were summarized with mean and standard deviation (SD). Frequencies and percentages were presented for categorical variables. Multivariate logistic regression analysis was performed to identify the associated factors to the occurrence of anastomotic leakage. Factors such as age, gender, location of tumor, histological subtype, comorbidity, pathological TNM stage were included in the multivariate logistic regression analysis. Mann-Whitney U test was performed to discover the impact of leakage to the length of hospital stay. Spearman rank correlation analysis, Mann-Whitney U test and Kruskal-Wallis test were performed to find out the influential factors to the length of hospital stay. Probability value of <0.05 was considered to be statistically significant. Statistical analysis was performed by using SPSS software (version 21) for Windows (SPSS Inc® headquarter, Chicago, IL, USA). This study was approved by the Ethics Committee of the Sun Yat-sen University Cancer Center (No. B2019-028-01) and informed consent was obtained from each patient.

## Results

Details of the clinicopathologic characteristics of patients and their correlations with anastomotic leakage are presented in Table 1.

**Table 1:** Clinical and pathological characteristics and their correlation with intrathoracic anastomotic leakage.

**Variables**	**No leakage**	**Leakage**	***p-*value**
Age (years, mean ± SD)	60.0 ± 9.0	62.0 ± 9.0	0.043^a,b^
Male, *n* (%)	907 (78.3%)	78 (79.6%)	0.42^a^
**Comorbidity**, ***n*****(%)**			
Hypertension	147 (12.7%)	11 (11.2%)	0.637^a^
Diabetes mellitus	29 (2.5%)	7 (7.1%)	0.015^a,c^
Hypertension & Diabetes	21 (1.8%)	4 (4.1%)	0.224^a^
**Histologic type**, ***n*****(%)**			
Squamous cell carcinoma	1,061 (91.5%)	90 (91.8%)	0.144^a^
Adenocarcinoma	38 (3.3%)	6 (6.1%)	0.091^a^
Other	60 (5.2%)	2 (2.0%)	0.282^a^
**Tumor location**, ***n*****(%)**			
Upper thoracic esophagus	40 (3.5%)	4 (4.1%)	0.765^a^
Middle thoracic esophagus	673 (58.1%)	50 (51.0%)	0.114^a^
Lower thoracic esophagus	442 (38.1%)	44 (44.9%)	0.286^a^
Pathological TNM stage, *n* (%)			0.182^a^
0	15 (1.3%)	1 (1.0%)	
I	157 (13.5%)	11 (11.2%)	
II	467 (40.3%)	31 (31.6%)	
III	453 (39.1%)	53 (54.1%)	
IV	24 (2.1%)	2 (2.0%)	
**Preoperative treatment**, ***n*****(%)**			
Chemotherapy	50 (4.3%)	4 (4.1%)	0.835^a^
Radiotherapy	0 (0%)	0 (0%)	
Chemoradiotherapy	5 (0.4%)	0 (0%)	0.615^a^

*SD, standard deviation*.

a *By Multivariate Logistic Regression analysis*.

b *OR(Odds Ratio): 1.024; CI(Confidence Interval):1.001-1.048*.

c *OR: 2.952; CI: 1.239-7.033*.

Totally 1,257 patients underwent esophagectomy with Sweet procedure between 2010 and 2015. Nine hundred and eighty-five (78.4%) patients were males. Two hundred and nineteen patients (17.4%) had comorbidities (Hypertension and/or Diabetes).

Overall squamous cell carcinoma was the dominant histological diagnosis of these patients (*n* = 1,151, 91.6%). Adenocarcinoma constituted only 3.5% of all cases. The other types included small cell carcinoma, neuroendocrine carcinoma and malignant melanoma, etc. Most tumors were located in the middle and lower part of esophagus (*n* = 1,213, 96.5%). The majority of patients were in stage II (*n* = 498, 39.6%) or stage III (*n* = 506, 40.3%).

Intrathoracic anastomotic leakage occurred in 98(7.8%) of 1,257 patients after esophagectomy. The results of multivariate analysis of factors related to anastomotic leakage are presented in Table 1.

The data showed that older patients were more likely to develop anastomotic leakage (*p* = 0.043). Patients with diabetes mellitus were associated with higher rate of anastomotic leakage (*p* = 0.015). Gender, hypertension, histologic type, tumor location and pathological TNM stage had no relevance to anastomotic leakage.

The length of hospital stay of patients after esophagectomy, and their correlation with anastomotic leakage are shown in Table 2.

**Table 2:** The effects of intrathoracic anastomotic leakage on hospital stay.

**Variables**	**No leakage**	**Leakage**	***p-*value**
Postoperative hospital stay (days, mean ± SD)	11.3 ± 5.3	41.1 ± 24.3	0.000^a^
Total hospital stay (days, mean ± SD)	17.6 ± 6.7	48.0 ± 24.6	0.000^a^

a *By Mann-Whitney U test*.

Mean length of total hospital stay was 17.6 days for patients without anastomotic leakage and 48.0 days in patients with anastomotic leakage (*p* = 0.000). Mean length of hospital stay after surgery was 11.3 days for patients without anastomotic leakage and 41.1 days in patients with anastomotic leakage (*p* = 0.000). From the above data, we could see that anastomotic leakage had a significant impact on the patient's hospital stay, it could increase the length of hospital stay by two to four times.

We further investigated the effects of different factors on postoperative hospital stay and total hospital stay, the results of statistical analysis are shown in Table 3.

**Table 3:** The effects of different factors on postoperative hospital stay and total hospital stay.

**Variables**	**Postoperative hospital stay**	**Total hospital stay**
	**(*p*-value)**	**(*p*-value)**
Age	0.000	0.001^a^
Gender	0.709	0.115^b^
Comorbidity	0.077	0.001^c^
Histologic type	0.732	0.933^c^
Tumor location	0.463	0.171^c^
Pathological TNM stage	0.211	0.206^a^

a *By Spearman rank correlation analysis*.

b *By Mann-Whitney U test*.

c *By Kruskal-Wallis test*.

The data demonstrated that age had a significant effect on patients' hospital stay, older patients were likely to stay longer in hospital (*p* = 0.000 or *p* = 0.001). Comorbidities could also prolong the total length of hospital stay (*p* = 0.001). Gender, tumor location and pathological TNM stage were not statistically associated with lengthened hospital stay.

## Discussion

Anastomotic leakage is a serious complication after radical resection of esophageal cancer. It is one of the important factors leading to the death of the patients and the decline of the quality of life. For a long time, the selection of the location of the anastomosis has been controversial. Although Walther and others believed that cervical anastomosis was as safe as intrathoracic anastomosis, and there was no difference in the incidence of anastomotic leakage (12, 13). However, most of the literatures suggest that cervical anastomosis is associated with a higher incidence of leakage (10–25%) than those performed in the chest (<10%) (14, 15). The present study summarized the incidence of intrathoracic anastomotic leakage (7.7%), which was similar to those reported literatures, and was lower than that of the cervical anastomotic leakage.

The aging of the population increases the number of elderly patients year by year. The incidence and mortality of complications in elderly patients may be higher than those of young people. The main reason may be the gradual decline of the physiological reserve function and the decrease of stress ability of the elderly patients. On the other hand, elderly patients are often accompanied by a variety of acute and chronic diseases. In addition, the elderly often suffer from malnutrition, anemia and lower immune function. All of these factors may lead to an increase in the incidence of anastomotic leakage in the elderly patients. The conclusion of our study is consistent with the cervical anastomotic literature (16), which also considered age as a risk factor for anastomotic leakage.

Diabetes is a systemic disease, due to long-term hyperglycemia status in the body's tissues and organs, a series of severe chronic pathological changes may take place in the heart, the kidneys, the retina, microcirculation, and the peripheral nerves, which in turn may hinder the healing of anastomosis. Recent literature from western country has reported similar conclusions, suggesting that diabetes mellitus and cervical anastomosis are risk factors for anastomotic leakage (17).

Once the anastomotic leakage occurs, it takes a relatively long time to treat and recover. Our study demonstrated that anastomotic leakage could significantly prolong the patient's hospital stay.

After examining the effects of some factors on the occurrence of intrathoracic anastomotic leakage, we continued to explore their influence on hospital stay. Among all the studied factors, we discovered that the age and commordity of patients had a significant impact on length of hospital stay. Older patients or patients with commordity (including hypertension and/or diabetes) would have longer hospital stay. This reminded us that we should be aware of the potential risks during the treatment of this sort of patients.

## Conclusion

In conclusion, our study suggested that old age and diabetes were risk factors to intrathoracic anastomotic leakage. In-hospital stay would be lengthened by intrathoracic anastomotic leakage, old age as well as comorbidity.

The present study was conducted on a relatively large number of patients compared to many other studies; however, the main limitation of this study is that it is single-center and retrospective. As far as “comorbidity” is concerned, this study only included hypertension and diabetes in the analysis and discussion. Further studies with prospective and multicenter design and more observed factors will better clarify the predictive factors for intrathoracic anastomotic leakage and some short-time postoperative outcomes.

## Data Availability Statement

The original contributions presented in the study are included in the article/supplementary material, further inquiries can be directed to the corresponding authors. The key raw data of this article have been uploaded to Research Data Deposit (www.researchdata.org.cn), with RDD number RDDA2021002120.

## Ethics Statement

The studies involving human participants were reviewed and approved by Ethics Committee of the Sun Yat-sen University Cancer Center (No. B2019-028-01). Written informed consent for participation was not required for this study in accordance with the national legislation and the institutional requirements.

## Author Contributions

QS and HL designed the study, applied for ethical approval and reviewed, edited, and finalized the manuscript. HL, SZ, HY, and WW collect the data. HL and SZ wrote the original manuscript. All authors contributed to the article and approved the submitted version.

## Conflict of Interest

The authors declare that the research was conducted in the absence of any commercial or financial relationships that could be construed as a potential conflict of interest.

## Publisher's Note

All claims expressed in this article are solely those of the authors and do not necessarily represent those of their affiliated organizations, or those of the publisher, the editors and the reviewers. Any product that may be evaluated in this article, or claim that may be made by its manufacturer, is not guaranteed or endorsed by the publisher.
